# Forecasting cancer incidence and prevalence using age–period–cohort and survivorship models: a practical, flexible, and interpretable framework

**DOI:** 10.3389/fonc.2025.1484896

**Published:** 2025-03-28

**Authors:** Ana F. Best, Adalberto M. Filho, Philip S. Rosenberg

**Affiliations:** ^1^ Division of Cancer Treatment and Diagnosis, Biometric Research Program, National Cancer Institute, Bethesda, MD, United States; ^2^ The International Agency for Research on Cancer (IARC), Lyon, France; ^3^ Division of Cancer Epidemiology and Genetics, National Cancer Institute, Bethesda, MD, United States

**Keywords:** breast cancer, forecasting, estrogen receptor, cancer incidence, cancer prevalence, age period cohort, joinpoint

## Abstract

Age–period–cohort (APC) model outputs have been used extensively to produce forecasts of cancer incidence, identify emerging public health concerns, and quantify the impact of potential interventions. However, these models have not been extended to forecast cancer *prevalence*—the number of cancer survivors per capita. Recent advancements in cancer screening and therapeutics have substantially improved survival for many malignancies, leading to an increased need to gauge the future health resource needs of cancer survivors. Concurrent shifts in cancer incidence trends require new methods to identify the separate and joint impacts of incidence and survival changes. In this paper, we formalize methods for forecasting incidence and introduce novel forecasting methods for prevalence that are highly flexible and interpretable. Our approach has three steps. First, we model cancer incidence trends by age, period, and birth cohort using the New APC Model. Second, we model all-cause mortality by age at diagnosis and year of diagnosis using flexible regression splines. Third, we estimate cancer prevalence as the convolution of cancer incidence and all-cause mortality, accounting for the need for backward projection of incidence to estimate prevalence during early periods. We illustrate our methods using data on invasive female breast cancer, stratified by estrogen receptor status, based on 1992–2019 SEER data. Our analysis illustrates how to calculate the relative impact of period vs. cohort effects on future incidence trends, the contributions of incidence trends and survival trends on future prevalence trends, and total case count estimation.

## Introduction

Age–period–cohort (APC) models provide an essential tool for modeling cancer incidence rates in populations (1). Age effects in the APC model describe the underlying age-associated cancer natural history; period effects quantify the impact of factors that affect all age groups simultaneously, e.g., changes in diagnostic practice; and cohort effects characterize net changes in incidence from one birth year to the next.

It is widely recognized that outputs from APC models can produce forecasts of cancer incidence (2). The underlying construction is especially straightforward using the Fundamental Decomposition Principle of the New APC Model. In essence, this approach makes predictions under the assumptions that the natural history stays the same; the birth cohort effects for observed cohorts hold steady; and plausible changes in incidence attributable to *future* periods and *younger* birth cohorts can be obtained by extrapolation from recent period and cohort trajectories, respectively. Such cancer incidence forecasts have identified emerging public health problems and provide a means to quantify the potential impact of future interventions (3–6). Other incidence forecasting methods include applying fixed incidence rates to population projections (7); extrapolating delay-adjusted average annual percentage change (8); Bayesian APC analyses (9); spatiotemporal models considering variation in sociodemographic, lifestyle, and health-related factors (10); and machine learning algorithms (11).

While APC models for incidence projection are established, we introduce novel methods to rigorously forecast cancer *prevalence*—the number of cancer survivors per capita over time by age group—under the APC structure. In recent decades, many types of malignancies have seen substantial improvements in therapeutic outcomes due to advances in treatment (e.g., targeted therapy and immunotherapy) and early detection via screening is well accepted for neoplasms of the colon, rectum, cervix uterus, and breast (12). In turn, this has increased the number of people in the population whose cancers have been cured, are in remission, or are manageable as long-term chronic diseases. Other work has established methods for estimation and projection of prevalence using the Mortality Incident Approach Model, which uses relative overall survival and a Weibull mixture cure model for survival extrapolation, and thus assumes constant survival rates during the projected period (13, 14). Other researchers have used linear regression models (15) or stock-and-flow models and observed prevalence proportions (16). It is of particular interest to establish APC-based prevalence forecasting methods due to the flexibility and interpretability of APC models and their widespread use in cancer surveillance research.

Forecasting cancer prevalence makes it possible to estimate the size of the cancer survivor community in a population over time and gauge their current and future healthcare needs (17). Indeed, cancer survivors typically require follow-up imaging, physician visits, and medications, and they may be affected by prolonged or delayed side effects of their cancer therapy, including second cancers and organ damage. Prevalence forecasts allow for anticipation of such needs up front rather than as a *post-hoc* reaction.

APC prevalence forecasts also extrapolate and incorporate temporal trends for survival, allowing for a much more comprehensive evaluation of prevalence trends than existing methods; under our novel model, it is possible to disentangle “conflicting” signals between incidence and prevalence rates in the population. In the absence of advances in treatment, cancer incidence and prevalence rates move in parallel: Increases in prevalence are a consequence of increases in incidence; both of these patterns indicate an adverse trend in the population. However, advances in treatment decouple the trajectory of prevalence from incidence. Moving forward, we anticipate many scenarios where incidence rates are declining, yet survival rates are increasing so rapidly that prevalence rates are increasing. Such scenarios represent simultaneous progress on two fronts, yet identifying these “double positives” will be difficult without suitable modeling tools.

Finally, counterfactual analysis within the framework of prevalence forecasting has several potential applications, including the identification of disparities in cancer care. In this report, we present a unified framework for modeling cancer incidence and prevalence by combining APC models for cancer incidence with flexible models for survival after cancer. Our frequentist approach is computationally light, provides appealing and easy-to-interpret outputs, and permits extensive scenario analyses. We will illustrate our new approach by forecasting female breast cancer prevalence by estrogen receptor (ER) status.

## Data and methods

### Invasive female breast cancer incidence by ER status

ER-positive (ER+) breast cancers are characterized by the presence of ERs in the tumor cells; tumors are typically characterized as ER+ if at least 1% of cells are positive via immunohistochemical (IHC) assay and comprised roughly 80% of breast cancers diagnosed during 2015–2019 (18).

In addition to traditional chemotherapy regimens, ER+ tumors may be treated with targeted endocrine therapies such as aromatase inhibitors (e.g., letrozole), selective ER modulators (e.g., tamoxifen), and selective ER degraders (e.g., fulvestrant).

Until relatively recently, there was no targeted therapy for ER− tumors (chemotherapy and radiation were the standard of care). Approximately 27% (19) of hormone-receptor-negative breast cancers overexpress *ERBB2*; such tumors are denoted HER2+ and may be targeted with several therapies. The first of these was the monoclonal antibody trastuzumab, approved by the Food and Drug Administration (FDA) for treatment of metastatic HER2+ breast cancer in 1998 (20); trastuzumab plus chemotherapy was approved for adjuvant therapy of nonmetastatic HER2+ tumors in 2006 (21). Subsequent research has refined the standard of care and introduced new targeted therapies including additional monoclonal antibodies (e.g., pertuzumab), tyrosine kinase inhibitors (e.g., lapatinib), and antibody–drug conjugates (e.g., ado-trastuzumab) (22). Treatment for triple-negative breast cancer continues to rely on cytotoxic chemotherapy, although early-stage tumors now receive neoadjuvant chemotherapy (23).

Interestingly, ER+ tumors have been increasing in incidence over time, whereas ER− tumors have been decreasing. Therefore, beyond a certain point, increases in the prevalence of ER+ tumors might reflect advances in therapy, i.e., good news. Conversely, at some point, any slowing of the rate of decrease in the prevalence of ER− tumors might also represent good news, as targeted therapies extend lives.

Given the high incidence of breast cancer in the US population, divergent incidence trends in ER+ versus ER− tumors, advances in the standards of care for both tumor types, and concomitant risks of long-term side effects of therapy, this disease remains of particular interest for forecasting both incidence and prevalence.

### Incidence and survival data

Our analysis was based on two sets of raw data for each tumor type: a cancer incidence Lexis diagram formatted for APC analysis (24) and a matching case listing of individual patients’ survival data (follow-up time and status at the end of follow-up) including age at diagnosis and year of diagnosis.

**Figure 1:**
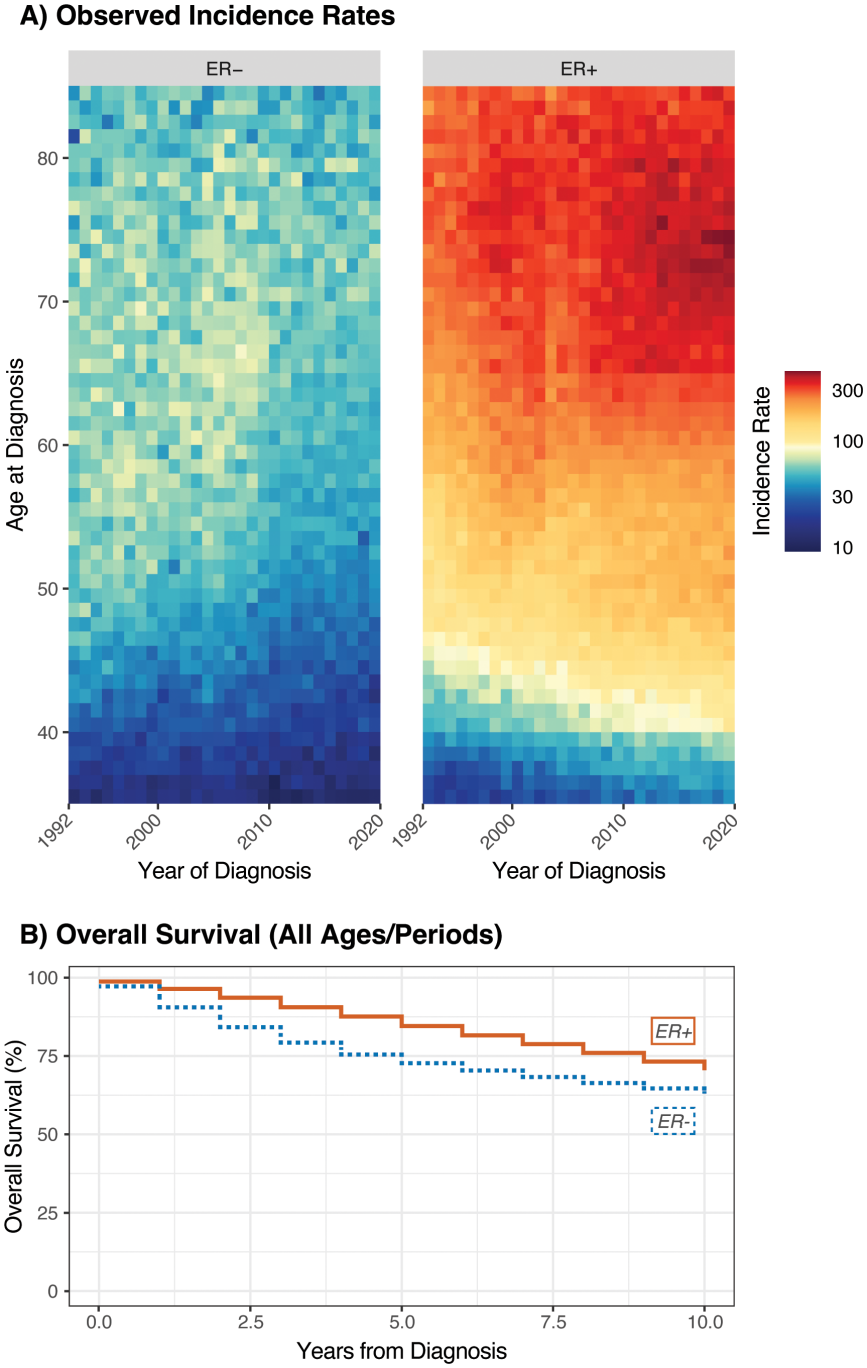
Lexis diagrams **(A)** and overall survival **(B)** for invasive female breast cancer by ER status; Ages 35-84, 1992-2019.

In our analysis, we obtained both types of data from the Surveillance, Epidemiology, and End Results (SEER) Program’s 12-Registry Database. Data were obtained for female patients with invasive breast cancer diagnosed between the ages of 30 and 84 (inclusive) and between 1992 and 2019; 2020 was excluded from both data sets due to the effects of the COVID-19 pandemic. Cases and rates were stratified by ER status due to the major differences in patterns of incidence, etiology, prognosis, and clinical management between these subtypes (25). Incidence rates were obtained by single year of age and year at diagnosis; survival case records were obtained by 5-year age group and exact year at diagnosis. From the incidence data, 2,087 (0.3%) cases with unavailable ER status and 60,031 (9.4%) cases with borderline/unknown ER status were excluded, with 578,827 (90.3%) incident cases remaining. From the survival data, 1,697 (0.3%) cases with unavailable ER status and 52,481 (9.4%) with borderline/unknown ER status were excluded, with 501,770 (90.3%) remaining. Lexis diagrams and a summary of overall survival are provided in **Figures 1A, B**, respectively.

Downstream forecasting of incident and prevalent case counts is possible by multiplying the estimated and forecast rates by estimates and projections for the corresponding underlying population. For forecasting using SEER, the US Census intercensal population estimates and forecasts are suitable for these purposes (26).

**Figure 2:**
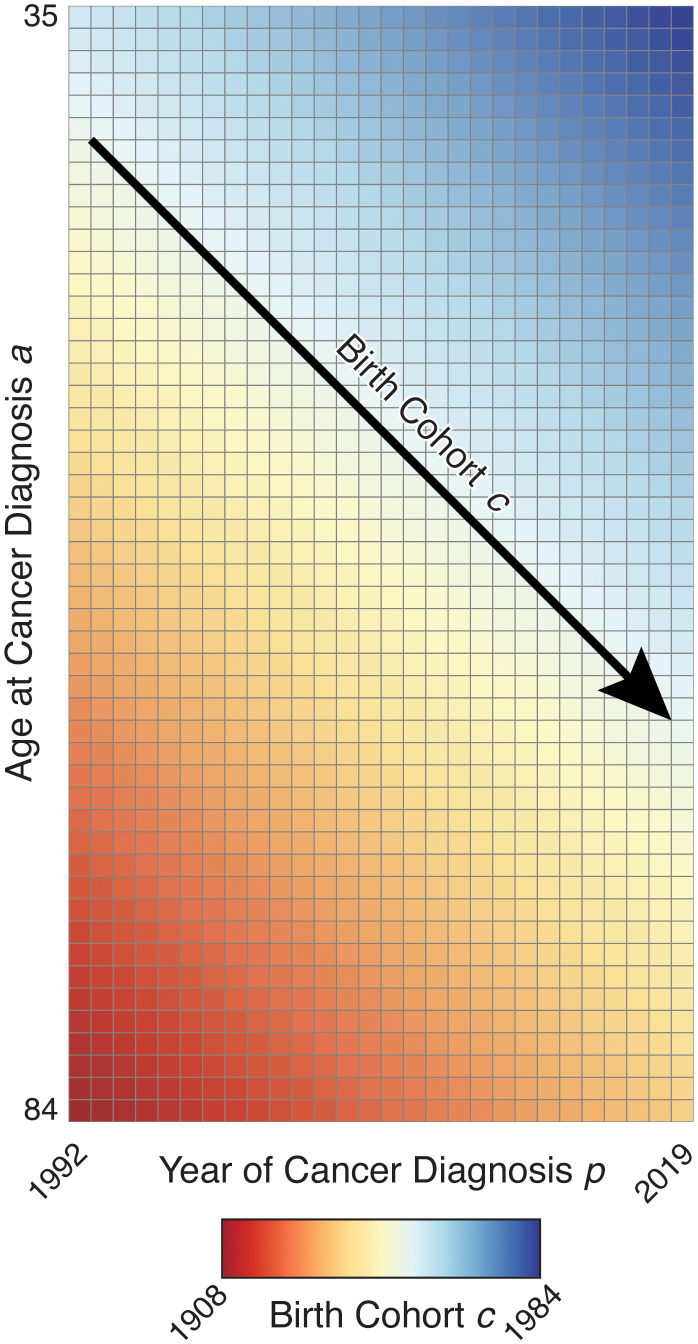
Illustration of Lexis Diagram.

We will use the following notation. For incidence, we have matrices $Y\;=\;\left[Y_{pa},\;p=p\left(1\right),\;\ldots ,\;p\left(P\right);\;a=a\left(1\right),\;\ldots ,\;a\left(A\right)\right]$ and $O=\left[O_{pa},\;p=p\left(1\right),\ldots ,p\left(P\right);\;a=a\left(1\right),\ldots ,\;a\left(A\right)\right]$ , which contain, respectively, the number of cancer diagnoses and corresponding person-years in period p and for age group *a*, for each of P periods and A age groups; the bin widths for age and period must be equal (common value Δ) . Then, birth cohorts form the diagonals, indexed by c=p−a , in order from oldest to youngest. The observed incidence rates are $\lambda_{pa}=Y_{pa}/O_{pa}$ and expected log rates are $\rho_{pa}=\text{ln}\left(E\left[Y_{pa}\right]/O_{pa}\right)$ . The Lexis diagram is illustrated in **Figure 2**, using ages and periods concordant with the breast cancer example.

**Figure 3:**
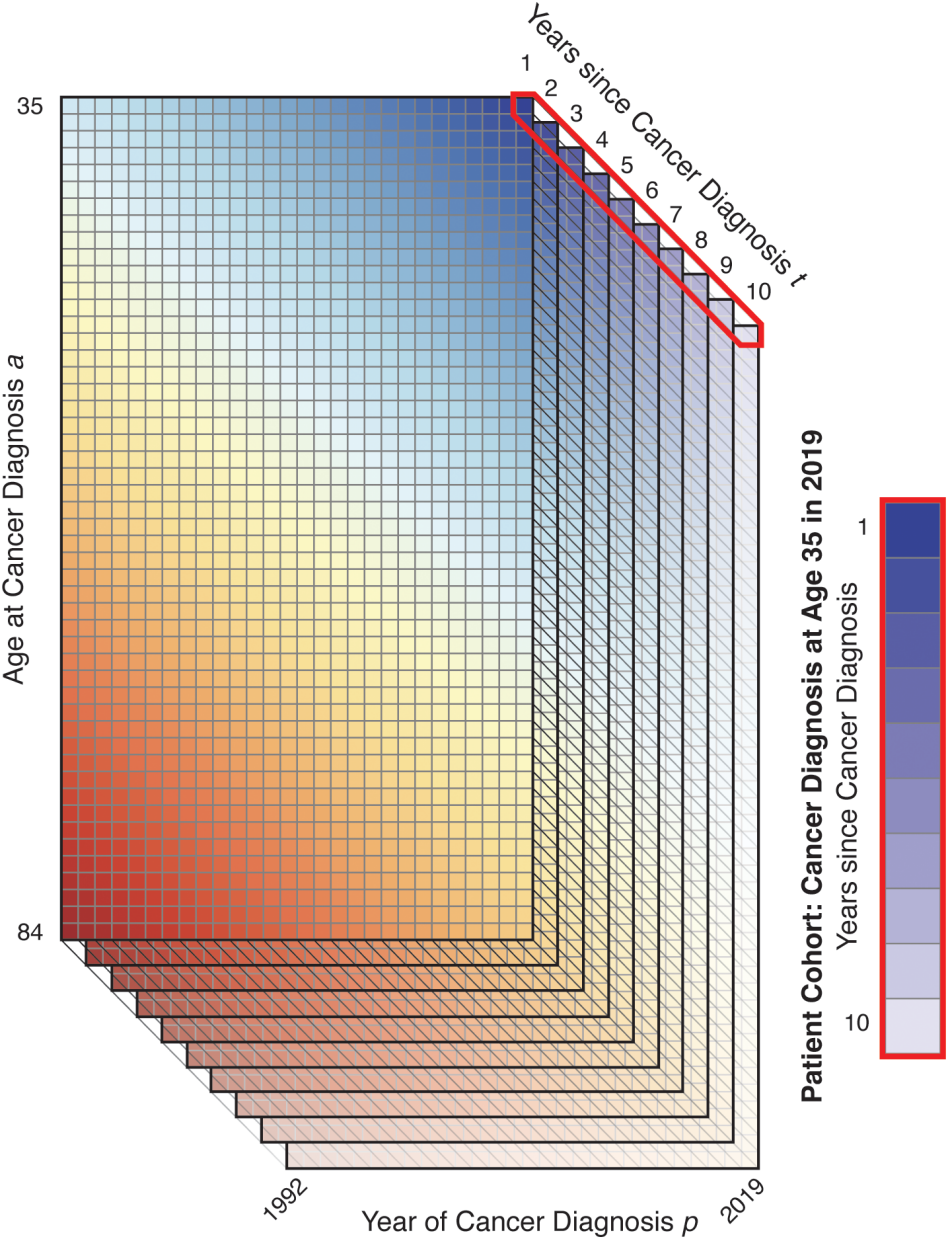
Illustration of Survivorship Matrix.

For survivorship, starting with the individual patient records, we tabulate these raw data into two three-dimensional matrices M and D , where $M_{a,p,t}$ is the number of persons who were diagnosed with their malignancy at age a in period p and who have survived to period p+t , and $D_{a,p,t}$ is the corresponding number of persons who died from *any cause* in period p+t ; for a=a(1),…,a(A) , p=p(1),…,p(P) , and t=t(0),…,t(T) , where t(0) means an individual died in the same period as they were diagnosed and t(T) is the maximum survival time considered to be part of the prevalent cohort (**Figure 3**). We count all-cause rather than cause-specific survival: patients who die are removed from the prevalent cohort whether or not their deaths are cancer-related.

With the data thus aggregated, we employ the New APC Model to estimate expected incidence rates and a discrete-time survival model to estimate the corresponding all-cause mortality rates, as described below. Notably, while the survival data should span the observed periods, it is not necessary for the size of the age groups and periods to match each other, or indeed to match those used for incidence rate forecasting. For example, survival data with 5-year age groups may be used to estimate prevalence alongside a rate matrix with single-year intervals for age and period, or *vice versa*.

### Incidence rate forecasting

Our forecasts rely on the New APC Model, built around a log-linear relationship between the incidence rate and age, period, and cohort: $\rho_{pa}=\alpha_{a}+\;\pi_{p}+\;\gamma_{c}$ , with the constraint that c=p−a . This may be reparametrized in several ways; for our projections, we will use both the longitudinal age–cohort form:

$\rho_{ca}=\mu +\left(\alpha_{L}+\pi_{L}\right)\left(a-\bar{a}\right)+\left(\pi_{L}+\;\gamma_{L}\right)\left(c-\bar{c}\right)+{\tilde{\alpha }}_{a}+\;{\tilde{\pi }}_{c+a}+{\tilde{\gamma }}_{c},$

and the cross-sectional age–period form:


$$\rho_{pa}=\mu +\left(\alpha_{L}-\gamma_{L}\right)\left(a-\bar{a}\right)+\left(\pi_{L}+\;\gamma_{L}\right)\left(p-\bar{p}\right)+\;{\tilde{\alpha }}_{a}+{\tilde{\pi }}_{p}+{\tilde{\gamma }}_{p-a}.$$

In these expressions, μ is the grand mean; ${\tilde{\alpha }}_{a},\;{\tilde{\pi }}_{p}$ , and ${\tilde{\gamma }}_{c}$ are the “complete” age, period, and cohort deviations, respectively; $\left(\alpha_{L}+\pi_{L}\right)$ and $\left(\alpha_{L}-\;\gamma_{L}\right)$ are the longitudinal and cross-sectional age trends; and $\left(\pi_{L}+\gamma_{L}\right)$ is the net drift. The New APC Model partitions the complete deviations into orthogonal quadratic and higher-order terms:


$${\tilde{\alpha }}_{a}=\;\theta_{\alpha }q_{a}^{2}\left(a\right)+\;{\overset{ˇ}{\alpha }}_{a};$$

$$\;{\tilde{\pi }}_{p}=\;\theta_{\pi }q_{p}^{2}\left(p\right)+\;{\overset{ˇ}{\pi }}_{p};$$

$$\;{\tilde{\gamma }}_{c}=\;\theta_{c}q_{c}^{2}\left(c\right)+\;{\overset{ˇ}{\gamma }}_{c}.$$

To obtain parameter estimates from this model, we further assume that the rates follow a Poisson or Quasi-Poisson distribution, and use weighted least squares or Poisson regression to estimate the parameters.

The Fundamental Decomposition Principle of the New APC Model allows us to rearrange the model parameters in several ways to express the absolute rates R(a,p) as a product of three estimable functions (EFs), one function each for age, period, and cohort, respectively. These decompositions use the following EFs: defining the incidence rate given period or cohort as R(a|⋅) ; the longitudinal and cross-sectional age curves at a selected reference cohort/period: $LongAge\left(a|c_{0}\right)=\text{exp}\left(\mu +\left(\alpha_{L}+\pi_{L}\right)\left(a-\bar{a}\right)+\;{\tilde{\alpha }}_{a}\right)$ and $CrossAge\left(a|p_{0}\right)=\text{exp}(\mu +\left(\alpha_{L}-\gamma_{L}\right)\left(a-\bar{a}\right)+\;{\tilde{\alpha }}_{a}$ ); and the cohort/period rate–ratio curves $CRR\left(c|c_{0}\right)=\text{exp}\left(\left(\pi_{L}+\gamma_{L}\right)\left(c-\bar{c}\right)+\;{\tilde{\gamma }}_{c}\right)$ and $PRR\left(c|c_{0}\right)=\exp\left(\left(\pi_{L}+\gamma_{L}\right)\left(p-\bar{p}\right)+\;{\tilde{\pi }}_{p}\right)$ . In what follows, we will use the following decompositions:


$$R\left(a|c\right)=LongAge\left(a|c_{0}\right)\times CRR\left(c|c_{0}\right)\times \exp\left(\theta_{\pi }q_{p}^{2}\left(p\right)+\;{\overset{ˇ}{\pi }}_{p}\right),$$

$$R\left(a|p\right)=CrossAge\left(a|p_{0}\right)\times PRR\left(p|p_{0}\right)\times \exp\left(\theta_{c}q_{c}^{2}\left(c\right)+\;{\overset{ˇ}{\gamma }}_{c}\right)$$

that provide two overall projection forms with numerous options. Under the age–cohort form, our forecasts are defined by the longitudinal age curve, the cohort rate ratio, and potentially the global curvature for period. The age–period form uses the cross-sectional age curve, period rate ratio, and global curvature for cohort.

**Figure 4:**
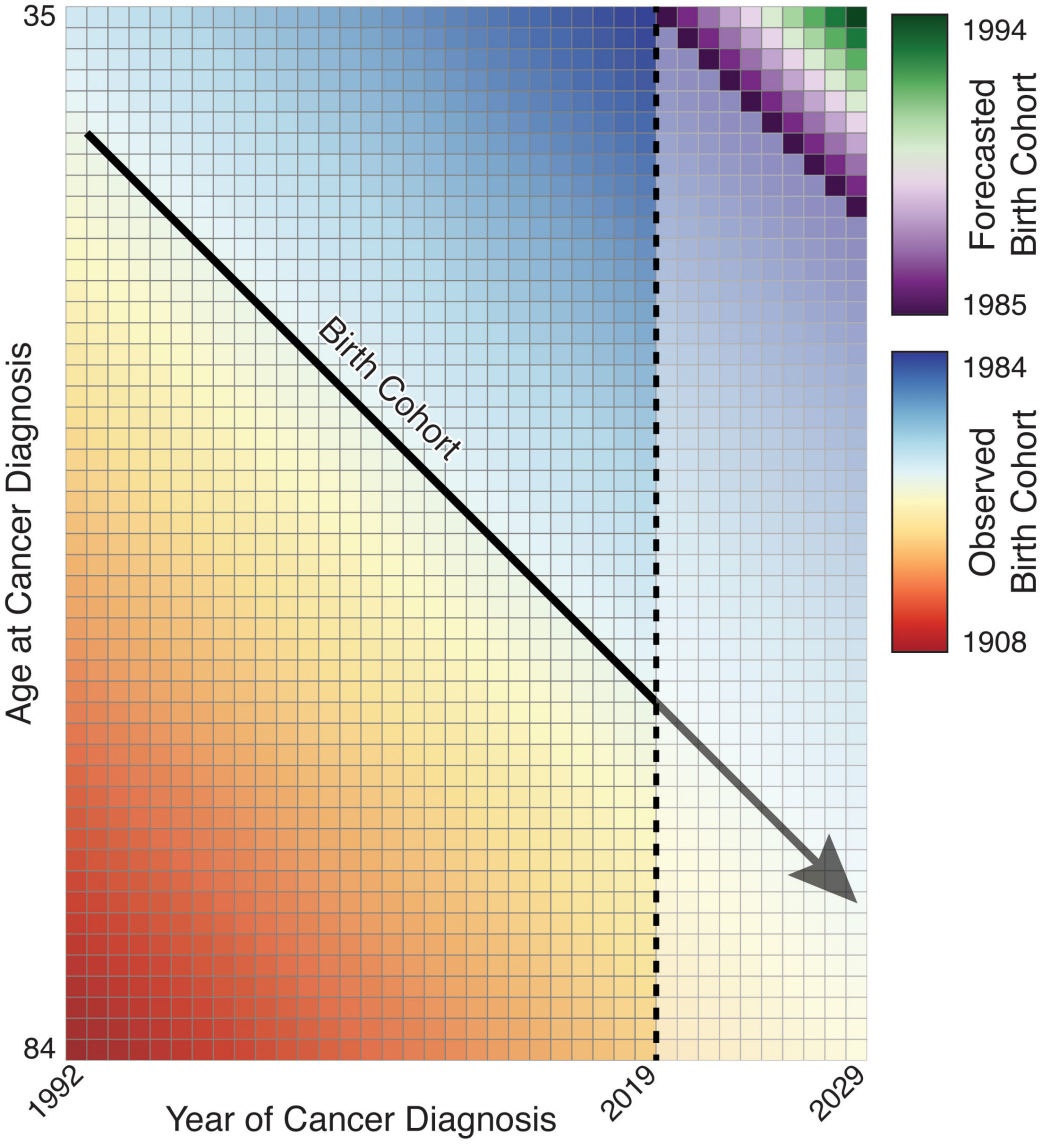
Illustration of birth cohort projection; partially observed birth cohorts are extended while unobserved birth cohorts must be forecasted.

**Figure 5:**
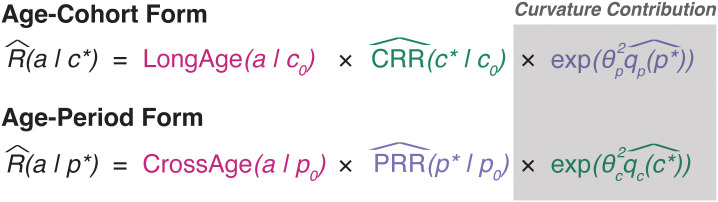
Incidence forecasting model forms.

Projections are built from these equations as follows. Under each form, the estimated age curve is assumed to hold during projected periods and is used without alteration. For age–cohort forecasting, forecasted cells corresponding to partially observed birth cohorts incorporate the estimated rate ratio for that cohort, and cells for unobserved birth cohorts use a linear extrapolation of a JoinPoint fit to the CRR values (**Figure 4**). Age–period forecasts use a linear extrapolation of a JoinPoint fit to the PRR for all forecasted cells. We may also choose to include components reflecting observed trends in the third variable, as the exponentiated slope of $\theta_{\pi }q_{p}^{2}\left(p\right)$ or $\theta_{c}q_{c}^{2}\left(c\right)$ at the last period or cohort, respectively (partially observed cohorts under the age–period form are forecasted using their fitted deviations). This provides four basic forecasting models for projected cohorts $c^{*}$ and periods $p^{*}$ (**Figure 5**).

Variances are calculated using the estimated variances from the APC and JoinPoint regression models. Additionally, this method may be used to forecast periods in both the future and the past; the latter will be required for prevalence estimation. The four models (or any subset thereof) may be combined into a single average of models using the mean of the estimates for each projected cell; variances may be estimated as the mean of the variances for each cell, or confidence intervals (CIs) estimated as the combined range of the modeled CIs. In addition, the fitted values and their covariances can be used to make plots that aggregate over any age groups and/or calendar periods of interest.

**Figure 6:**
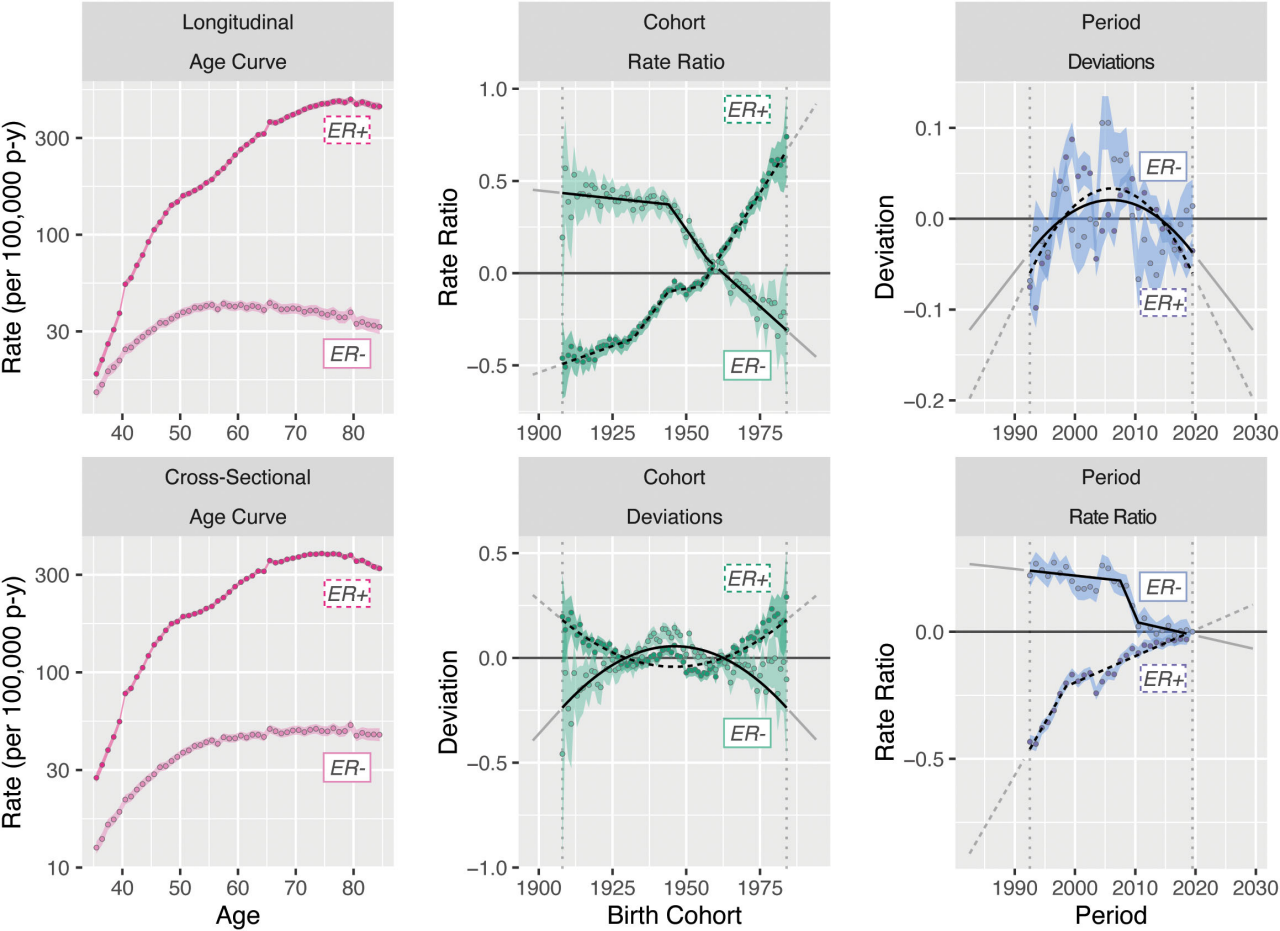
Incidence model decomposition for invasive female breast cancer by ER status.

**Figure 6** illustrates our two decompositions and extrapolations for breast cancer by ER status. The top panels correspond to the age–cohort form and the bottom panels correspond to the age–period form. Estimated rates have been aggregated for ages 35–49 and 50–84 to correspond, respectively, to premenopausal women who are not routinely screened for breast cancer and postmenopausal women who undergo mammography screening.

**Figure 7:**
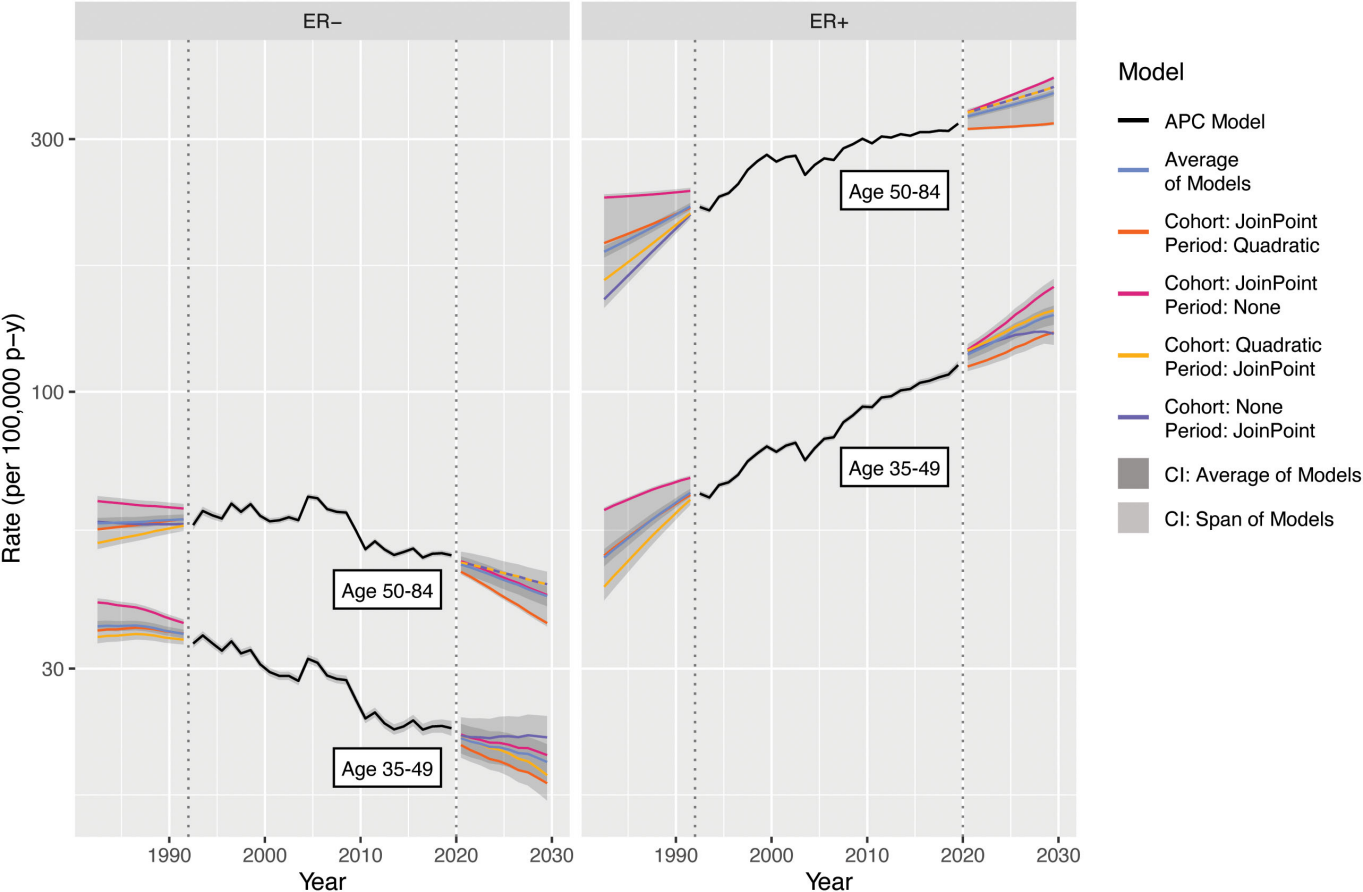
Incidence forecasts for breast cancer by age and model. Fitted values and covariances from models for ages 35-84 are used to aggregate estimates for ages 35 49 and 50-84.

Incidence forecasts are obtained by multiplying the age–incidence curve by the fitted or forecast cohort and/or period components as appropriate for the forecasted cell. **Figure 7** illustrates four forecasts based on different extrapolations, as well as the average of models, by ER status and age. Rates and variances were summarized within age groups using a linear operator (1). The two age–period model curves for 2020–2030 closely coincide as the majority of birth cohorts during this period contributed to the observed data.

Examining the 2020–2030 forecasts, the extrapolated quadratic period effects reduce estimated incidence. Without this component, forecasts are predominantly influenced by the observed changes in CRR; ER+ rises more steeply and ER− falls less steeply for both age groups. The period–JoinPoint models are driven by more moderate changes in PRR; the extrapolated cohort effects are applied only to the youngest cohorts in the projection, which consequently are of young age and have low absolute estimated incidence. The averaged forecast, with a CI covering the span of the models, provides a reasonable single forecast and summary of possible future incidence rates. The forecasts for 1982–1992 notably represent a projection of invasive breast cancer rates by ER status to periods during which this variable was not captured by the SEER registries, potentially allowing imputation of this variable for earlier SEER data.

### Prevalence rate forecasting

Estimation and forecasting of prevalence are of particular interest as this can be a difficult quantity to measure directly; cancer registries typically focus on incident cases, as these are substantially easier to capture. Prevalence by its nature requires a somewhat retrospective outlook—the cohort of cancer survivors each year is composed of not only people diagnosed that year but also those diagnosed in previous years who survived. Individuals with malignancies that require ongoing management, such as metastatic disease or those with a long period of adjuvant therapy, may be identifiable as active patients at cancer treatment centers. However, individuals who have been cured of their cancer may no longer routinely receive treatment at cancer centers but are still relevant to prevalent-disease cohorts. Although these individuals may not be in active treatment for their cancer, they may nonetheless be experiencing long-term side effects from their malignancy or its treatment and may also be in need of tailored surveillance due to the risk of developing secondary malignancies. Within registry data, the cohort of survivors also potentially includes those diagnosed prior to the start of registration, risking underestimation if not accounted for.

**Figure 8:**
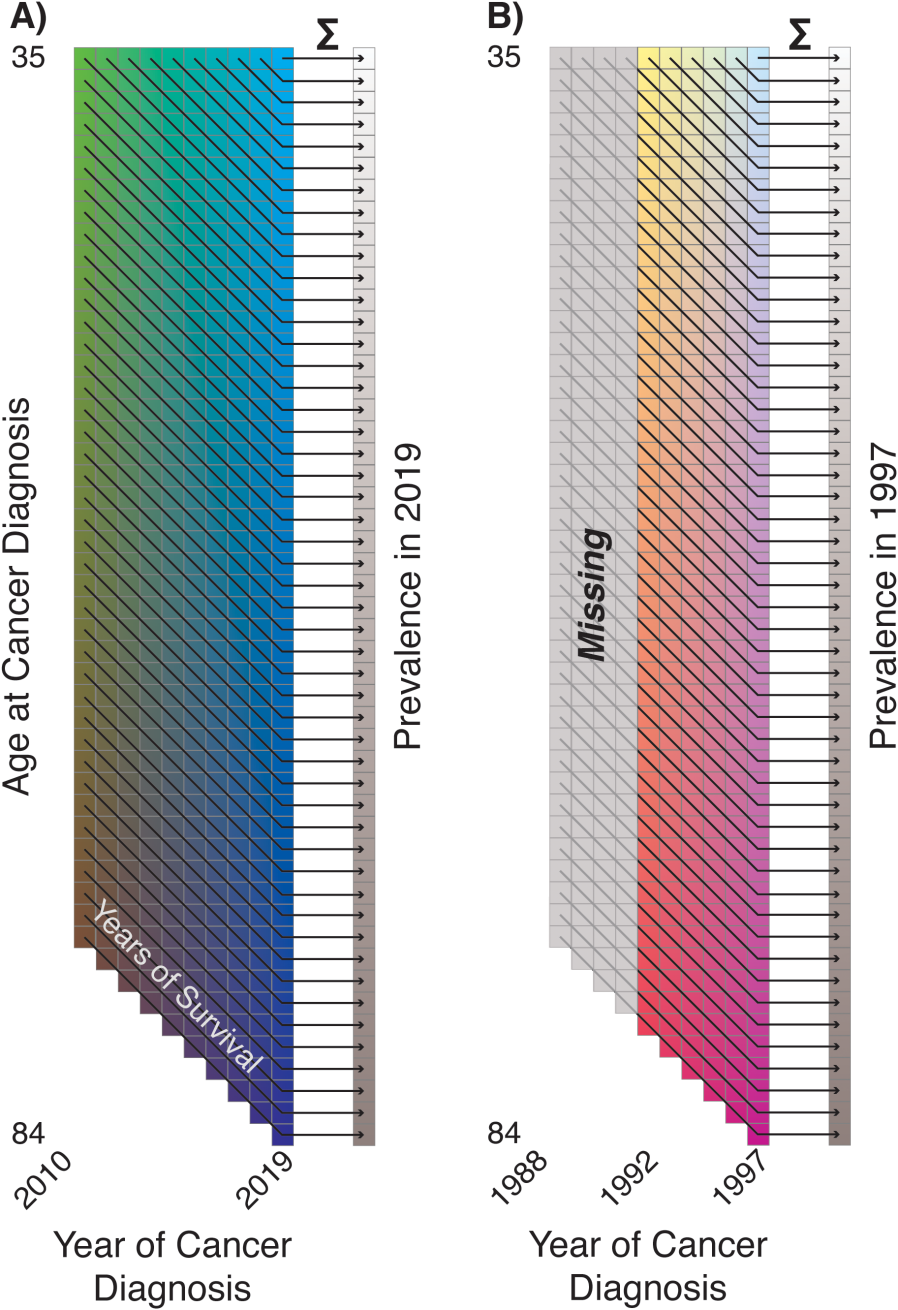
**(A)** Illustration of prevalence as convolution of incidence and survival; **(B)** illustration of incompleteness of early period prevalence estimates ('Burn-In').

We directly use this retrospective outlook on prevalence to calculate our estimates and forecasts, based on the fundamental principle that prevalence is a convolution of incidence and survival. If we are interested in the cohort of cancer survivors at age a(a) in year p(p) , this is composed of those diagnosed at age a in year p , those diagnosed at age a(a−1) in year p(p−1) who survived (at least) Δ years, those diagnosed at age a(a−2) in year p(p−2) who survived 2Δ years, and so forth (**Figure 8A**). This immediately prompts a “burn-in” problem: prevalence estimates are by default missing the convolution components from years prior to the first year of the incidence data (**Figure 8B**). This provides the justification for the backwards forecasting of incidence rates shown in the previous section: if we “forecast” incidence rates and survival trends into the past, those forecasts may be used to estimate the missing convolution components and provide prevalence estimates during the observed period. As a rule of thumb, if we define our prevalence cohort as containing individuals who have survived up to t(T) years after their cancer diagnosis, we must forecast t(T) years into the past to completely estimate prevalence; the choice of t(T) is an experimental design consideration depending on the survival distribution and number of years of available incidence data.

**Figure 9:**
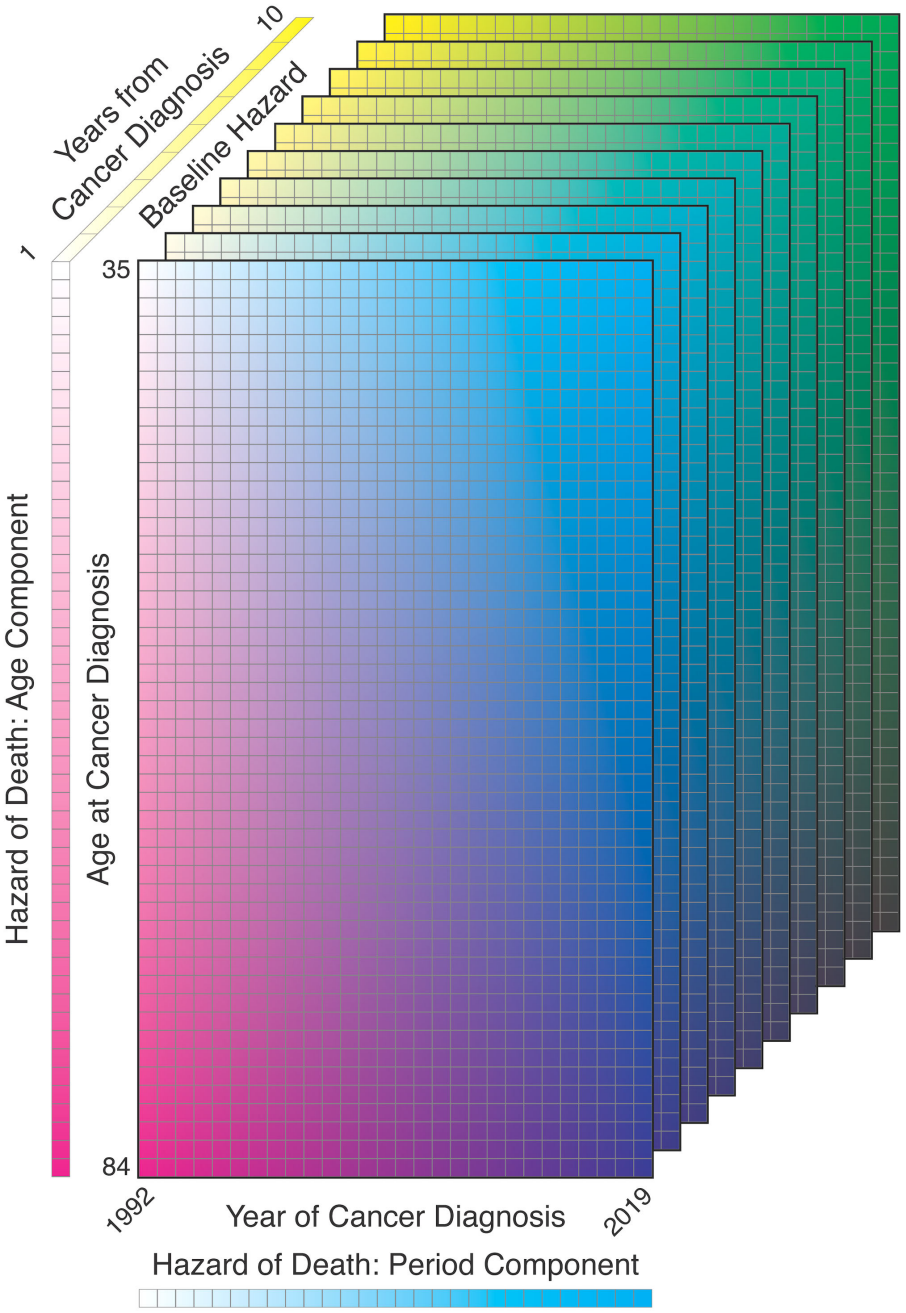
Illustration of survival model components: age at diagnosis, year of diagnosis, years from diagnosis.


Using the three-dimensional tabulated survival data represented by M and D , we can use a discrete-time survival model (the GAM approach) including terms for the baseline hazard $\nu_{T}\left(t\right)$ of *all-cause* survival for the malignancy of interest as well as effects for age $\left[\nu_{A}\left(a\right)\right]$ and period [ $\nu_{P}\left(p\right)]$ of diagnosis (**Figure 9**):

$$\ln\left(\frac{E\left[D_{a,p,t}\right]}{M_{a,p,t}}\right)=\nu_{a,p,t}=\nu_{T}\left(t\right)+\nu_{A}\left(a\right)+\nu_{P}\left(p\right).$$

These hazard terms are estimated using piecewise linear splines for all three components, as these are readily extrapolated:


$$\nu_{a,p,t}=\nu +\sum_{i=0}^{k_{T}}\delta_{i}{\left(t-\tau_{T,i}\right)}^{+}+\sum_{j=0}^{k_{A}}\psi_{j}{\left(a-\tau_{A,j}\right)}^{+}+\sum_{k=0}^{k_{P}}\phi_{k}{\left(p-\tau_{P,k}\right)}^{+}.$$

In the above, ${\left(\cdot \right)}^{+}$ indicates inclusion of only positive summands, i.e., components where s, a , or p is higher than the corresponding τ value. The three linear splines are defined on partitions $0=\tau_{T,0}<\;\tau_{T,1}<\ldots <\tau_{T,k_{T}}=T$ , $a\left(1\right)=\tau_{A,0}<\;\tau_{A,1}<\ldots <\tau_{A,k_{A}}=a\left(A\right)$ , and $p\left(1\right)=\tau_{P,0}<\;\tau_{P,1}<\ldots <\tau_{P,k_{P}}=p\left(P\right)$ , with hazard contributions $\delta_{0},\ldots ,\delta_{k_{T}}$ , $\psi_{0},\ldots ,\psi_{k_{A}}$ , and $\phi_{0},\ldots ,\phi_{k_{P}}$ . Among other methods, these splines may be fit using JoinPoint, which estimates the number and location of the partition knots as well as the slopes, or B-Splines with fixed knots based on quantiles. As with incidence rate forecasting, the spline for period is extended linearly into past periods for “backfilling” prevalence and future periods for forecasting.

**Figure 10:**
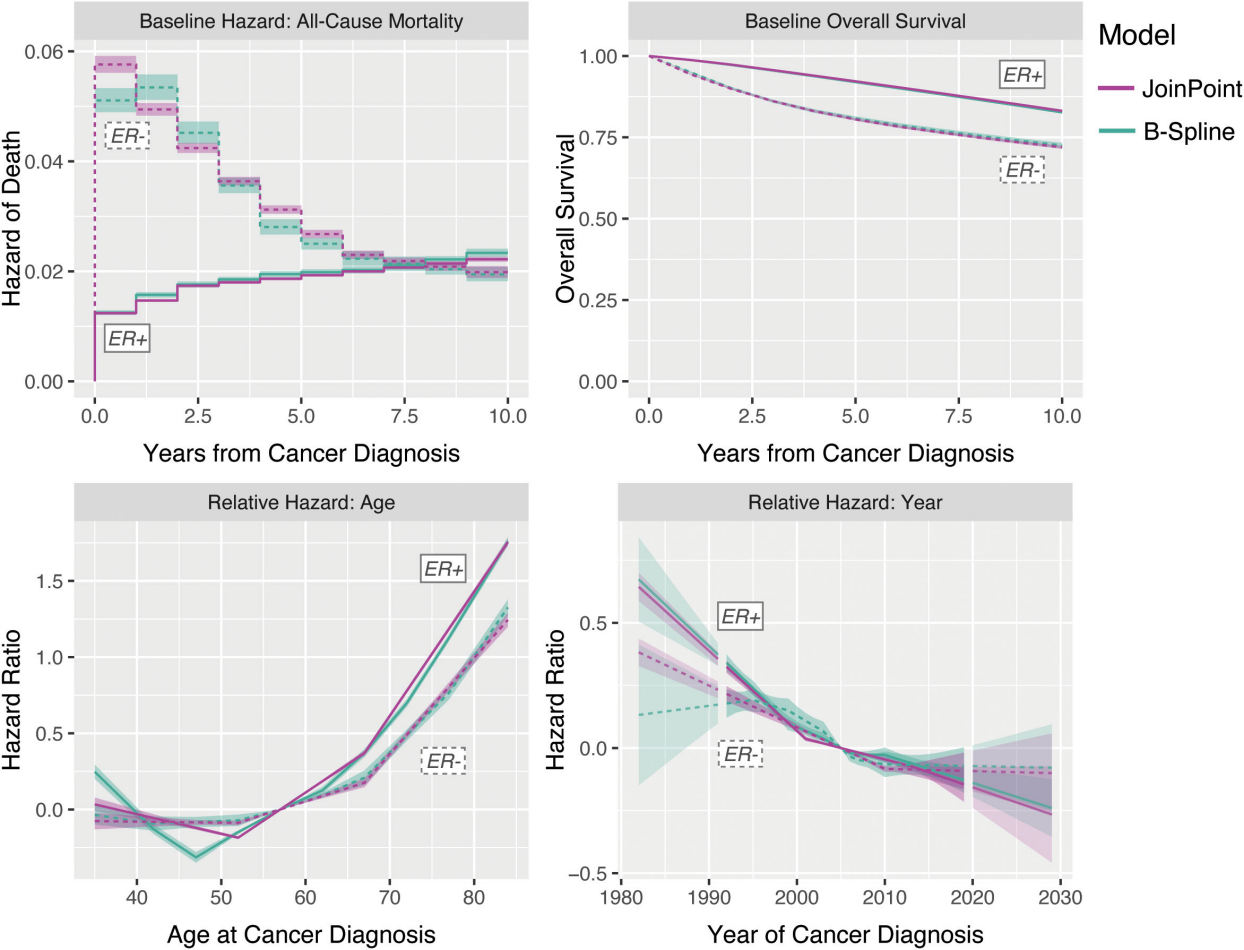
Survival model decomposition for invasive female breast cancer by ER status.

We used SEER case listing survival data for the ages and periods included in the incidence rate forecast, censored at t(T)=10 years post-diagnosis. In these data, age is available only in 5-year groups and was imputed as the midpoint of each age group for analysis purposes; extrapolating the fitted splines provides survival trend estimates for all ages under consideration. **Figure 10** illustrates the survival model estimates and extrapolations by ER status, using both JoinPoint and B-Splines; the estimates are largely concordant between the two estimation methods, except for the B-Spline and JoinPoint hazard ratios for ER− due to differences in the placement and number of knots relative to the JoinPoint. This figure shows the trends we would expect for these malignancy subtypes: overall good survival, with increasing hazard of death for older age at diagnosis and relatively steady decreasing hazard of death with more recent year of diagnosis.

We may now formally combine these components; we define a matrix $S\;=\;\left[S_{pa},\;p=p\left(1\right),\;\ldots ,\;p\left(P\right);\;a=a\left(1\right),\;\ldots ,\;a\left(A\right)\right]$ to be the number of prevalent cases at age a and period p , corresponding to the previously defined offset O . The prevalence rates are defined as $\xi_{pa}=S_{pa}/O_{pa}$ and expected log rates are defined as $\omega_{pa}=\text{ln}\left(E\left[S_{pa}\right]/O_{pa}\right)$ . $E\left[S_{pa}\right]$ can be calculated as:


$$E\left[S_{pa}\right]=\;\sum_{i=t\left(0\right)}^{t\left(T\right)}E\left[M_{a-i,p-i,i}\right]=\sum_{i=t\left(0\right)}^{t\left(T\right)}E\left[Y_{p-i,a-i}\right]\exp\left(-\sum_{j=t\left(0\right)}^{t\left(i\right)}\nu_{a-i,p-i,j}\right)$$

In practice, as the incidence and prevalence rates share an offset, this may be simplified to:


$$E\left[\xi_{pa}\right]=\sum_{i=t\left(0\right)}^{t\left(T\right)}E\left[\lambda_{p-i,a-i}\right]\exp\left(-\sum_{j=t\left(0\right)}^{t\left(i\right)}\nu_{a-i,p-i,j}\right)$$

Due to the multi-step estimation procedure, it is not straightforward to calculate closed-form variances; therefore, we will use a parametric bootstrap procedure. First, we sample b matrices of incidence rates from the matrix of Poisson distributions with rates λ . These sampled rate matrices may then be convolved with the estimated hazard array to obtain b matrices of sampled prevalence rates. The AP×AP -sized estimated covariance matrix is then calculated as the sample covariance of the AP×b matrix of sampled and estimated rates; the A×P estimated variance matrix is obtained from the diagonal.

**Figure 11:**
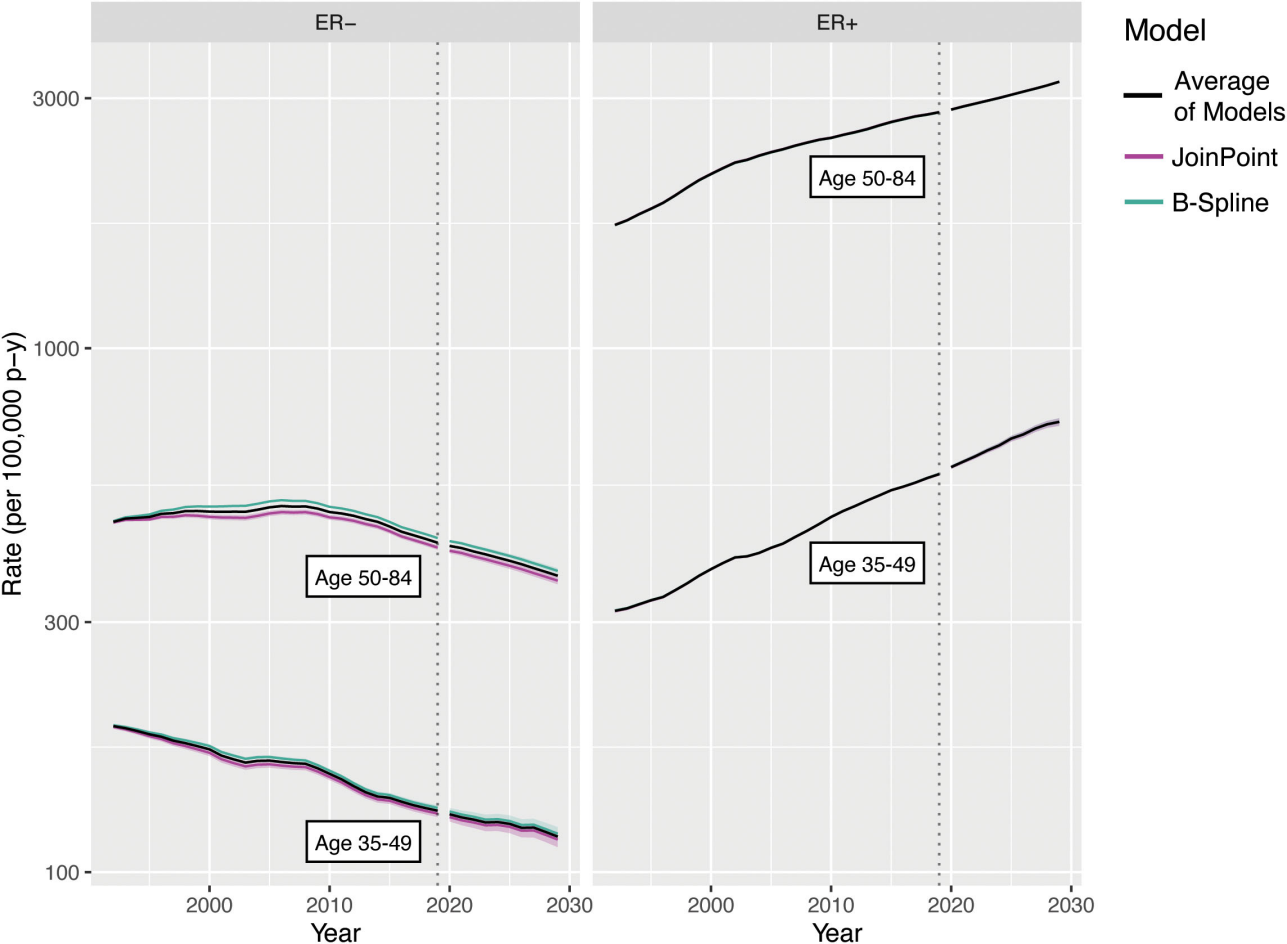
Survival rate forecasts for invasive female breast cancer by age and ER status. Fitted values and covariances from models for ages 35-84 are used to aggregate estimates for ages 35-49 and 50-84.

Applying this to breast cancer and averaging the results from the JoinPoint and B-Spline survival models, we obtain the estimated prevalence rates illustrated in **Figure 11**; concordant with **Figure 10**, the two models produce nearly identical estimates. The combination of increasing incidence rates and survival improvements results in increasing prevalence for ER+, while ER− prevalence is forecast to decline due to decreasing incidence.

### Counterfactuals, sensitivity analyses, and forecast decomposition

A primary strength of our model is its flexibility and allowance for easy incorporation of sensitivity analyses and other forms of counterfactuals. The incidence and survival forecasts are each generated using linear extensions of an observed trend, and can be directly replaced by another line, either predetermined by the analyst or derived in light of the observed estimates. For example, it is possible to evaluate scenarios with 0.5× or 1.5× the estimated rate of change by applying this multiplicative factor to the estimated linear extension. The linear extensions of the quadratic components of the deviations may also be replaced by the full quadratic curve, if concordant with the observed data.

**Figure 12:**
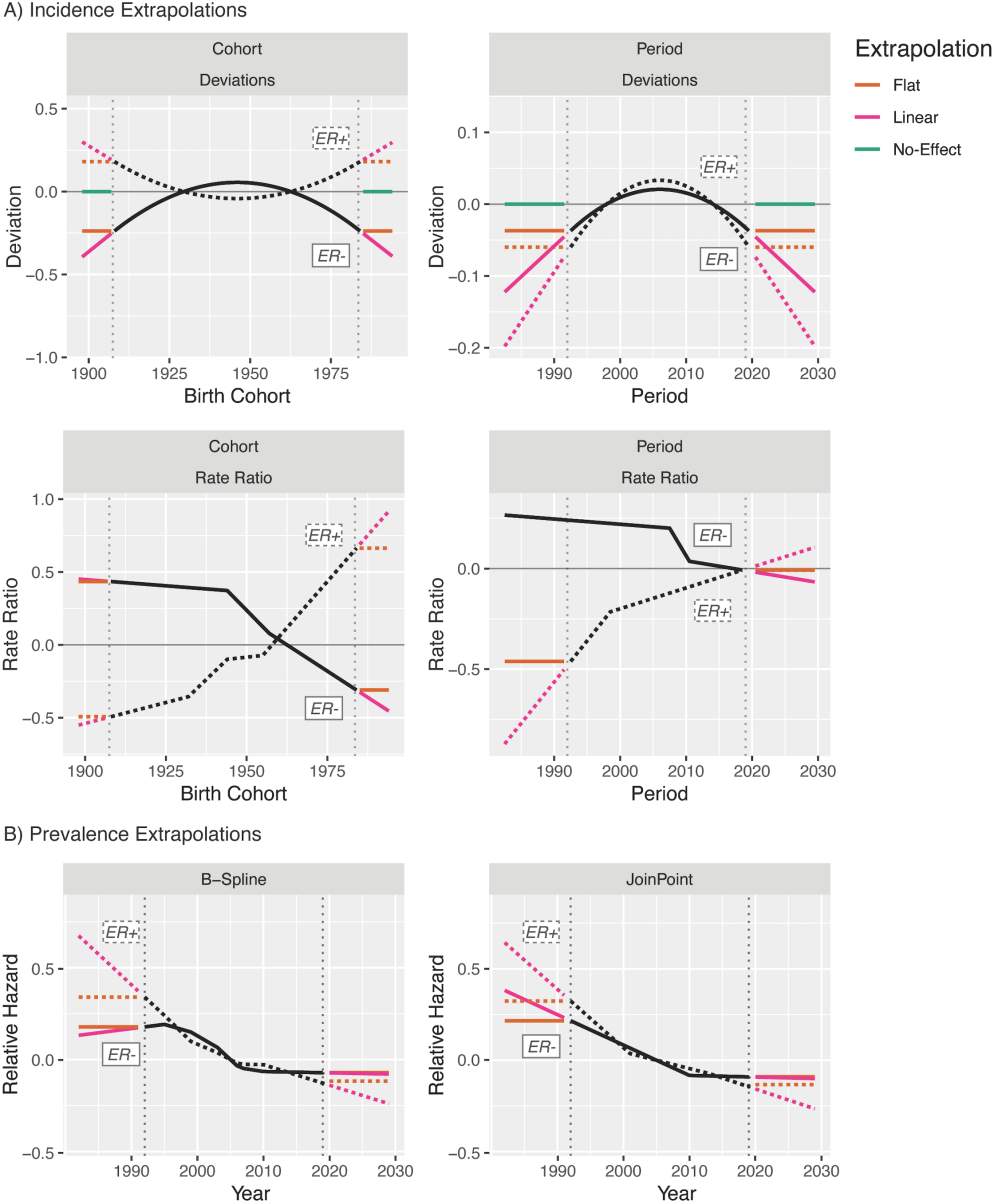
Extrapolation model options for invasive female breast cancer incidence **(A)** and survival **(B)**.

This flexibility allows us to decompose the forecasts and evaluate the specific contributions of birth cohort and period trends in incidence rates, and period trends in estimated survival, to the final forecasts. **Figure 12A** illustrates the extrapolation models used for this decomposition for breast cancer by ER status: Rate ratios and survival can be forecast as either a linear extension of the last linear spline component or a horizontal line extending from the last fitted linear spline value. Deviations can be forecast as the continuation of the quadratic fit (not pictured), a linear extension using the slope at the last period, a horizontal line extending from the quadratic at the last period, or with no contribution to the forecasts at all.

**Figure 13:**
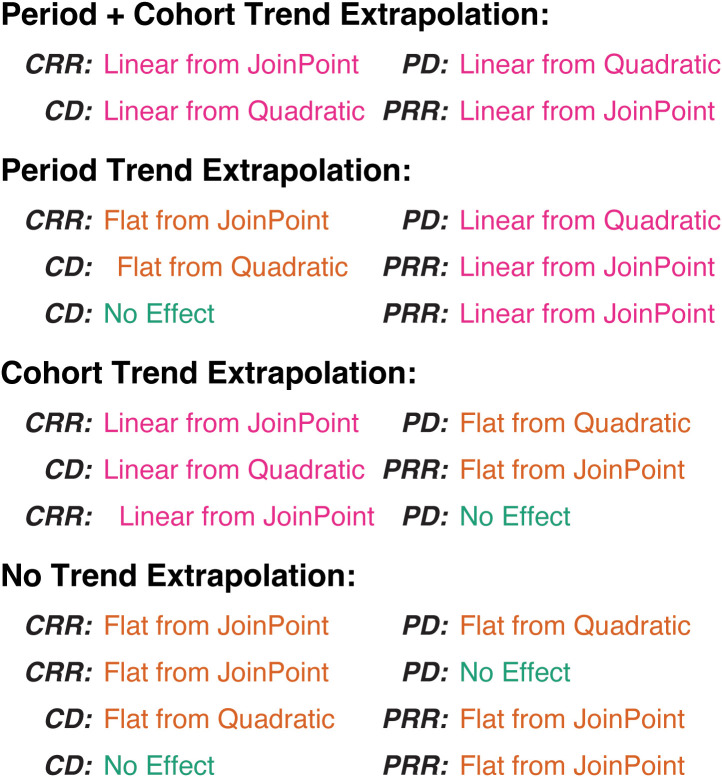
Summary of incidence forecasting models.

Based on these possibilities, we have 12 valid incidence forecasting models that may be categorized into four groups, those that extrapolate trends for both period and cohort, for either one, or for neither (**Figure 13**).

**Figure 14:**
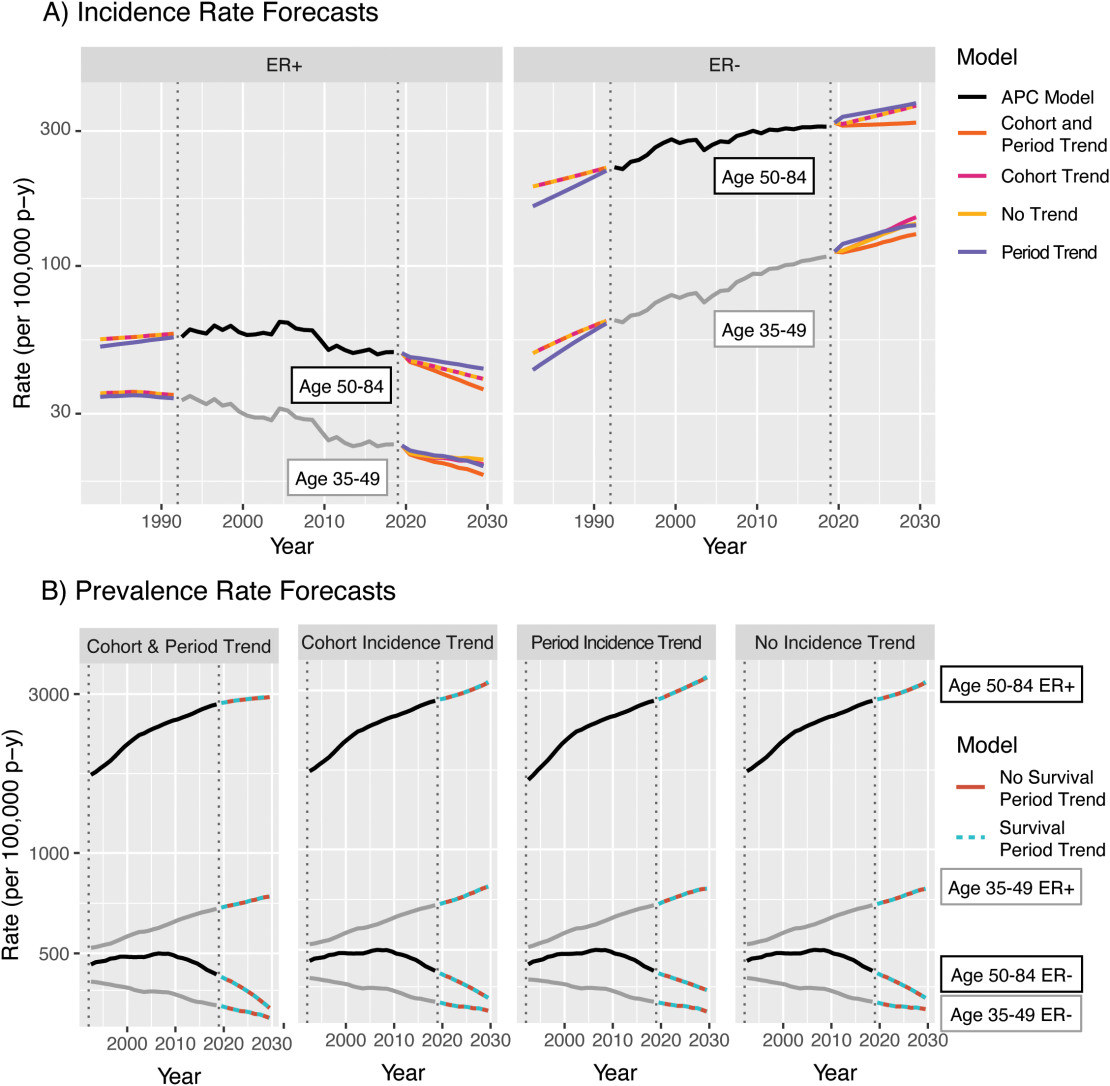
Averages of models for invasive female breast cancer incidence **(A)** and prevalence **(B)** by age group, ER status, and trend components.

The results from averaging the projections and their variances across these four sets for ER+ breast cancer are shown in **Figure 14A**. In this example, as in other examples where incidence rates are dramatically lower for young ages than old ones, the forecasts for models with no trend incorporated are nearly equivalent to those with period or cohort trends only; although **Figure 12A** shows strong forecasted cohort effects, these are only applied to completely unobserved birth cohorts, the oldest of which is aged only 44 in 2029. Inclusion of period effects in addition to cohort effect results in a lower overall forecast, driven by the strong downward quadratic trends in the period deviations. Evaluating the effect of the survival trend in **Figure 14B**, replacement of the period trend for survival by a horizontal extension of the 2019 hazard ratio only minimally affects forecasts as the estimated rate of change in the HR is relatively small for both subtypes.

### Case counts: burden and survivorship

**Figure 15:**
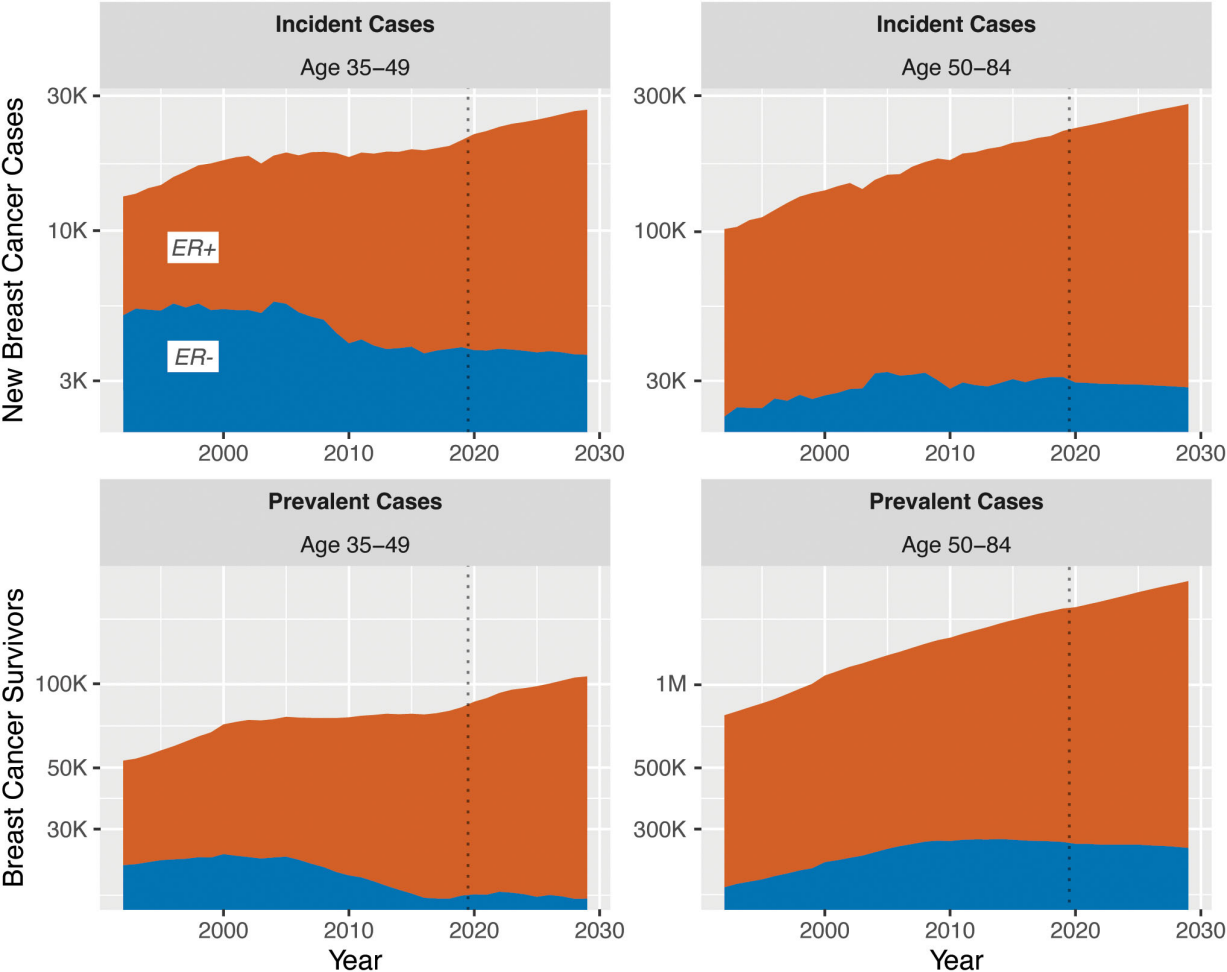
US Population burden and survivorship for invasive female breast cancer, by ER status and age group.

Forecasted rates may be applied to population estimates to obtain estimated annual incident case counts (burden) and the number of people living with a diagnosis within the last T years (survivorship). A natural set of population estimates to combine with SEER data are those from the United States Census, which provides decennial exact counts, intercensal estimates, and forecasts under multiple scenarios of immigration level. The results for ER+ and ER− breast cancer are shown in **Figure 15**; for both age groups, while the overall burden and survivorship are both projected to increase for ER+ disease, ER− count projections are steady.

## Discussion

Our new methods for projecting cancer incidence and prevalence have several advantages, both practical and conceptual. Perhaps the most important of these is its intermediate level of complexity. APC forecasting allows for more flexibility and nuance than simpler models, but does not require additional spatiotemporal data sources and is highly interpretable, serving as a “glass box” model in comparison to complex “black box” machine learning models. Each component of the forecasts may be viewed individually as in **Figures 6** and **10**; **Figure 12** illustrates the ability to evaluate the contributions of each component to the final forecast directly.

It is straightforward to evaluate counterfactual situations reflecting the influence of potential interventions or changes in standard of care on incidence and prevalence. APC forecasting is not computationally burdensome. Although JoinPoint models for a large number of birth cohorts can become computationally intensive, this can be compensated for by placing conservative bounds on the JoinPoint model parameters (e.g., the maximum number of knots or periods/cohorts per segment) and by memorizing fitted JoinPoints to avoid re-fitting them in multi-faceted analyses of the same incidence rates. Furthermore, averaging model estimates allows us to produce estimates free of strong *a priori* assumptions or *post-hoc* decisions regarding which model components might provide the strongest signals for forecasting, or whether JoinPoint or B-Spline models might provide the best fit to the survival data.

Forecasting shows us the future implications of current patterns. It makes sense to consider forecasts whenever the model goodness of fit (GOF) is adequate. Importantly, there is one model in play for incidence but two models determine prevalence. GOF for incidence can now be assessed using SAGE (1), and the APC models for incidence provide good fit to SEER breast cancer data for both ER subtypes. GOF for mortality can be assessed using classic tests of interactions in multiplicative models for cohorts (27). Development of formal model averaging methods is a potential area for future research as well.

APC forecasting also has several disadvantages and limitations. Our incidence models do not account for situations in which the age–incidence curve (either cross-sectional or longitudinal) changes shape rather than amplitude with varying periods and/or cohorts. Lack of fit can now be evaluated using the SAGE method (1, 3). When lack of fit appears to be substantial, one can fit models and construct forecasts to reduced sets of periods or ages. However, reducing the number of periods used for estimation also reduces the number of periods that may be forecasted reliably, and combining separate forecasts across age groups does not account for the overlap in birth cohorts between groups.

Extending the quadratic deviation components provides additional flexibility for incidence forecasting beyond that allowed by the JoinPoint fits to the rate ratios alone but may not provide a reasonable summary or plausible extension of the observed deviations. This may be evaluated by examining the model decomposition plots, but any compensation for poor fit would then be *post hoc*. Prevalence forecasting also requires forecasting incidence rates and survival trends into past unobserved periods, and a poor-fitting back-forecast may be carried forward to the rest of the forecast. If this is of particular concern, back-forecasting may be omitted and the first t(T) periods may be used instead as a “burn-in” period for which prevalence estimates are incomplete and not reported.

The biggest limitation of the model is shared by other forecasting paradigms: it is difficult to quantify the total uncertainty of the forecasts. Our methods produce variance estimates and CIs that reflect the uncertainty inherent in the model, under the assumption that the observed trends hold during the forecasted periods. However, as the COVID-19 pandemic has illustrated, it is impossible to foresee and account for all possible future events. **Figure 7** illustrates one alternate quantification method: uncertainty intervals for an average-of-models forecast may be calculated as the span of the model-uncertainty CIs of the averaged models. However, this limitation is primarily addressed in the interpretation of the forecasts: they reflect a “snapshot” of the future as determined by past and present trends, which may be affected by future events both intentional (e.g., interventions or changes in standard of care) and not (e.g., pandemics, disruptions in surveillance, or other unforeseen events). In practice, we would recommend that scientists using these methods fit multiple plausible prediction models; from these, the average may be reported as a point estimate and the range may be reported as a measure of uncertainty in the prediction.

In addition to introducing statistical methodology, our work illustrates several important trends in invasive female breast cancer. Survival for both subtypes has improved over 1992–2019, although ER− survival has been stable since roughly 2010, while ER+ shows continued slow but steady improvement since approximately 2000. Cohort JoinPoints for incidence indicate that rates of ER− cancer have been steadily declining in women born in approximately 1945 or later, while ER+ has been steadily increasing for women born since approximately 1955. Period JoinPoints show that ER− incidence has been stable or slightly declining since approximately 2010 and a decline in the rate of increase for ER+ beginning around 2010. Evaluating the forecasts, the effects of recent and future cohort birth cohort trends will be seen primarily in periods beyond those forecast by this model, owing to low absolute incidence rates among younger women. Prevalence rate forecasts for both subtypes are primarily driven by changes in incidence, as survival has been approximately stable in both groups during the last decade. To ensure reproducibility of our analyses, SEER data have been used without imputation of missing ER status values; ER missingness in these data is time-dependent (approximately 50% of patients with missing values were diagnosed prior to 2000) and trends for imputed data may differ slightly from those shown here. Further detailed evaluation of these trends and counterfactuals is an additional potential area of future research.

Overall, APC forecasting is a computationally tractable method of producing nuanced yet highly interpretable estimates of future incidence and prevalence rates, as illustrated here for breast cancer. APC incidence forecasting has been used in a variety of scientific settings, both within and outside of oncology, and we believe that the expansion of the APC toolbox to include prevalence forecasting is a valuable step forward for cancer epidemiology. The R code for these methods is built on that from the APC “toolbox” and is freely available from the authors upon request.

## Data Availability

The original contributions presented in the study are included in the article/supplementary material. Further inquiries can be directed to the corresponding author. All data are publicly available from the US NCI Surveillance, Epidemiology, and End Results (SEER) program and US Census Bureau.
